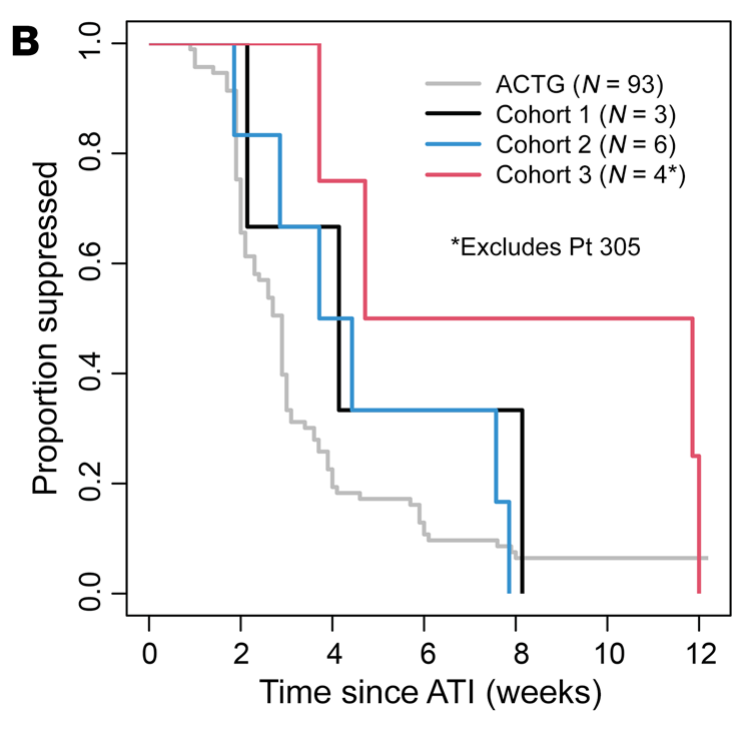# CCR5-edited CD4^+^ T cells augment HIV-specific immunity to enable post-rebound control of HIV replication

**DOI:** 10.1172/JCI181576

**Published:** 2024-05-01

**Authors:** Pablo Tebas, Julie K. Jadlowsky, Pamela A. Shaw, Lifeng Tian, Erin Esparza, Andrea L. Brennan, Sukyung Kim, Soe Yu Naing, Max W. Richardson, Ashley N. Vogel, Colby R. Maldini, Hong Kong, Xiaojun Liu, Simon F. Lacey, Anya M. Bauer, Felicity Mampe, Lee P. Richman, Gary Lee, Dale Ando, Bruce L. Levine, David L. Porter, Yangbing Zhao, Don L. Siegel, Katharine J. Bar, Carl H. June, James L. Riley

Original citation: *J Clin Invest*. 2021;131(7):e144486. https://doi.org/10.1172/JCI144486

Citation for this erratum: *J Clin Invest*. 2024;134(9):e181576. https://doi.org/10.1172/JCI181576

During the preparation of this manuscript, the third cohort shown in Figure 2B was mislabeled. The correct figure panel is shown below, and the HTML and PDF versions of the article have been updated online.

The *JCI* regrets the error.

## Figures and Tables

**Figure 2:**